# 160. Missed Opportunities: Gaps in Osteoporosis Screening Among Older Adults Living with HIV

**DOI:** 10.1093/ofid/ofaf695.055

**Published:** 2026-01-11

**Authors:** Rasha S Abdalla, Rachel Konchal, Shweta Kapur, Wiliam Carrington, Lauren Touleyrou

**Affiliations:** Wayne state University/ DMC, NOVI, MI; Wayne state University/ DMC, NOVI, MI; Wayne state University/ DMC, NOVI, MI; Wayne state University/ DMC, NOVI, MI; Wayne State University, Detroit, Michigan

## Abstract

**Background:**

Advancements in HIV care over the past 30 years have led to a significant increase in life expectancy. HIV infection is independently associated with decreased bone mineral density, and bone fractures occur approximately 10 years earlier in those with HIV. Certain antiretroviral therapies (ART), particularly those containing tenofovir disoproxil fumarate (TDF), are also associated with osteoporosis.

This study assessed DEXA scan screening rates and the prevalence of osteoporosis and osteopenia among people living with HIV (PLWH).

**Methods:**

A retrospective cohort study was conducted of 856 PLWH aged ≥ 50 who visited the Infectious Disease Clinic at least once in 2024. Data collected included the frequency of DEXA screening, the prevalence of osteopenia/osteoporosis, and history of TDF-based ART exposure. Descriptive statistics were calculated, and the distribution of the data was analyzed using the Shapiro-Wilk test.

**Results:**

Among 851 eligible HIV-positive patients (median age 61 years, IQR 56-66), 297 were female, 550 were male, and 9 were transgender female. Only 174 (20.5%) underwent DEXA scan screening. Of those screened, 62 (35.6%) had normal bone scans, 79 (45.4%) were diagnosed with osteopenia, and 33 (19.0%) were found to have osteoporosis. In the previous year, 553 out of 841 patients (65.8%) were on TDF-based regimens. Among those with osteoporosis, 20 (60.6%) had TDF exposure, while 50 (63.3%) patients with osteopenia received TDF-based treatments.

**Conclusion:**

Despite guideline recommendations for osteoporosis screening in PLWH aged ≥ 50, DEXA scan rates remain low. The study highlights the need for improved processes in ordering DEXA scans for PLWH, particularly those on TDF-based ART regimens. Implementing systematic changes to increase DEXA scan requests could lead to earlier detection of decreased bone mineral density and improved management of bone health in this population. Future efforts will focus on developing and integrating effective strategies to enhance screening rates and optimize patient outcomes.

**Disclosures:**

All Authors: No reported disclosures

